# The role of peer relationships and flow experience in the relationship between physical exercise and social anxiety in middle school students

**DOI:** 10.1186/s40359-023-01473-z

**Published:** 2023-12-06

**Authors:** Yao Shang, Shan-Ping Chen, Li-Ping Liu

**Affiliations:** Center for Physical Education, Xi’an Jiaotong University, Xi’an, 710049 China

**Keywords:** Physical exercise, Social anxiety, Peer relationships, Flow experience, Middle school students

## Abstract

The present study examined the relationship between physical exercise and social anxiety by testing a moderated mediation model that focused on how peer relationships mediate the relationship between physical exercise and social anxiety and how flow experience moderates this mediated relationship. A total of 1056 middle school students from six middle schools in Sichuan, China, volunteered to complete questionnaires comprising the Physical Activity Rating Scale, Student Peer Relationship Scale, Social Anxiety Subscale of the Self-Consciousness Scale, and Short (9-Item) Dispositional Flow Scales. Regression analysis indicated that physical exercise negatively influenced social anxiety through peer relationships (indirect effect = -0.04, 95% *CI* = [-0.067, -0.013]). In addition, a moderated regression analysis indicated that under high-flow experience, physical exercise suppresses social anxiety through positive effects on peer relationships (indirect effect = -0.04, 95% *CI* = [-0.087, -0.003]), and under low-flow experience, physical exercise exacerbates social anxiety through negative effects on peer relationships (indirect effect = 0.06, 95% *CI* = [0.016, 0.105]). Some practical implications have been discussed on the physical exercise intervention for suppressing social anxiety in middle school students.

## Introduction

Social anxiety refers to the negative emotional experiences that individuals have in social situations, such as worry, fear, and nervousness [1]. The development of social anxiety can lead to long-term tension in interpersonal interactions, reducing learning ability, social skills, and seriously endangering the physical and mental health of students [2]. Related studies have pointed out that middle school students in adolescence are a common population suffering from social anxiety symptoms [3] and the prevalence of social anxiety among them is increasing every year [4]. The effective prevention and treatment of social anxiety in middle school students hold significant public health implications. In recent years, physical exercise as an effective means of promoting individual physical and mental health, its mitigating effect on social anxiety has been widely studied by researchers, but the pathway of the effect of physical exercise on social anxiety is still unclear [5]. Therefore, this study aims to elucidate the relationship between physical exercise and social anxiety and provide a reference for promoting the mental health of middle school students.

The mitigating effect of physical exercise on social anxiety in individuals has been demonstrated [6]. Previous research has also shown that peer relationships are strongly associated with social anxiety [7], and students with poorer peer relationships tend to exhibit more avoidant behaviors in social situations [8], suggesting a negative effect of peer relationships on social anxiety. And physical exercise provides individuals with more opportunities to communicate and enhance peer relationships [9]. These pieces of evidence suggest a mediating role for peer relationships between physical exercise and social anxiety. However, previous studies have not focused on the relationship between physical activity, peer relationships, and social anxiety. In addition, the role of exercise experience is often overlooked by scholars, and Li et al. [10] noted that exercise experience may be a more important factor in the promotion of mental health by exercise compared to exercise volume. Specifically, positive exercise experiences are more likely to enhance interactions between individuals, which facilitates the establishment of individual peer relationships in physical exercise and promotes mental health. And the flow experience is a positive and ideal exercise experience that needs to be pursued during physical exercise [11], and research has confirmed that acquiring the flow experience helps to strengthen the relationship among exercise peers [12], suggesting that the acquisition of flow experience may facilitate the positive effects of physical exercise on peer relationships. Accordingly, in the present study, we focused on examining (1) the mediating role of peer relationships between physical exercise and social anxiety and (2) the role of flow experience as an ideal exercise experience on this mediating mechanism.

### Effect of physical exercise on social anxiety

In recent years, the positive influence of physical exercise on individual psychology has been discussed. Physical exercise is a physical activity with a certain intensity, frequency, and duration for the purpose of improving physical health [13]. From an emotional perspective, it has been shown that physical exercise releases catecholamines, which are associated with a pleasant and positive state of mind, contributing to the reduction of anxiety [14], and the effect of physical exercise on social anxiety has been suggested. An empirical study of 484 adolescents’ mental health may also provide evidence, as researchers found that students who exercised more had significantly lower levels of social anxiety than those who exercised less [5]. Based on the above evidence, there is reason to believe that physical exercise can be effective in reducing social anxiety in middle school students.

### Peer relationships as a mediator

Peer relationships have become a hot topic in positive psychology in recent years. It refers to an interpersonal relationship established and developed in the process of interaction between peers or individuals of comparable psychological development, with the functions of mutual recognition, shared happiness and shared fear [15]. In one study, peer relationships were found to be negatively related to social anxiety, and students who developed secure and stable relationships with their peers were better able to interact with others, strengthen interpersonal relationships, and were more relaxed in their later social activities and less prone to social anxiety [16]. Judge et al. [17] also noted that when individuals encounter frustration in the process of building peer relationships, they are more likely to perceive it as a reflection of their inadequacy in interpersonal skills, which further leads to the development of social avoidance behaviors. Furthermore, some scholars have made attempts to help students establish positive peer relationships. Among these methods, physical exercise is considered an effective way to build peer relationships. Chen and Yu [18] have pointed out that physical exercise promotes the consolidation and development of peer relationships, which is a basic motivation for individuals to participate in physical exercise. These pieces of evidence suggest that physical exercise may be conducive to the establishment of positive peer relationships and further limit the development of social anxiety.

### Flow experience as a moderator

Under what conditions can physical exercise better help students build peer relationships and reduce social anxiety? As an ideal state of internal experience, flow experience may be a variable worth considering. In the field of physical exercise, flow experience is a positive and ideal exercise experience and psychological state in which the participant is fully engaged, attentive, and feels impressed and full of value afterwards [11, 19]. Previous research has shown that exercise experience plays a moderating role in the effect of physical exercise on an individual’s body self-esteem, and the effect of physical exercise on an individual’s body self-esteem was maximized at a high level of exercise experience [20]. Moreover, cultivating high levels of body self-esteem can enhance students’ confidence in interpersonal interactions and facilitate the development of positive peer relationships [21]. This underscores the potential for the flow experience, as the optimal experience during physical exercise, to optimize the benefits of physical exercise for peer relationships. Additionally, in team sports, individuals who enter the flow experience are highly focused and less likely to be disturbed by external factors, which facilitates exercise participants to perform better in sports [22] and gain the trust of their peers, thus strengthening the bond between individuals and other team members. Based on this, the study concluded that individuals may have higher levels of peer relationships when they enter the ideal experiential state of flow experience in physical exercise. In other words, flow experience may play a moderating role between physical exercise and peer relationships.

**Fig. 1:**
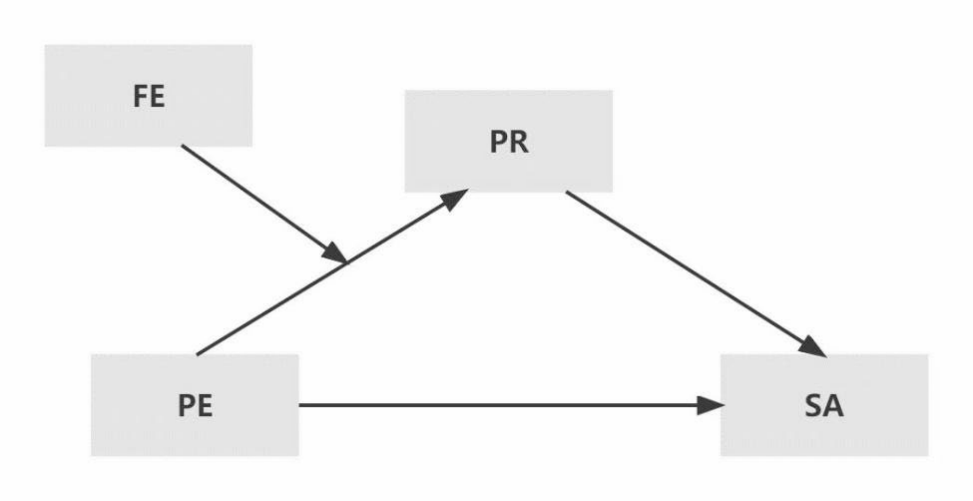
Theoretical model PE, physical exercise; PR, peer relationships; SA, social anxiety; FE, flow experience

In summary, we propose a moderated mediation model (as shown in Fig. 1) to systematically analyze the relationship between physical exercise and social anxiety in a sample of Chinese middle school students to provide a theoretical basis for further explaining the relationship between physical exercise and social anxiety, and to provide suggestions for improving social anxiety among middle school students from the perspective of physical exercise. Based on the existing literature, the following hypotheses were tested:

#### Hypothesis I

Physical exercise could negatively predict social anxiety.

#### Hypothesis II

Peer relationships played a mediating role between physical exercise and social anxiety.

#### Hypothesis III

Flow experience played a moderating role between exercise and peer relationships.

## Materials and methods

### Participants

The present study involved the participation of 1094 middle school students from six schools in Sichuan, China, selected through a random sampling approach. The sample size was determined according to standard rules of thumb for survey research, exceeding the questionnaire entries by a factor of 20 and taking into account an inefficiency rate of 10% [23]. Each participant filled out the survey questionnaire online according to the instruction ahead of the scale and their counselor from June 10, 2022, to July 28, 2022. After completing the survey, data preparation was carried out. If more than 70% of the same answers were chosen in a questionnaire, it was considered invalid. Consequently, 38 questionnaires were eliminated because they were incomplete or contained more than 70% of identical responses. In total, 1056 questionnaires were determined to be valid, resulting in a validity rate of 96.5%. The ages of the participants ranged from 11 to 15 years old (*M* = 13.28; *SD* = 1.08).

### Procedure

This study was approved by the Biomedical Ethics Committee of Xi’an Jiaotong University Health Science Center in accordance with the Declaration of Helsinki (No. 2022 − 1595). Participants and their legal guardians signed an informed consent form. The data collection process was initiated by sending an email to 21 counselors from six middle schools in Sichuan, China. The email contained two parts: the first part explained the purpose of the study and the conditions for recruiting participants (voluntary, anonymous, and confidential). The second part was a link to an online platform that participants were asked to complete with a consent form and measurement instrument items. The counselors then distributed the information in the email to the students through their class groups (due to the impact of the Covid-19 pandemic in the first half of the year, all students completed the study course through online devices during the pandemic, and each class formed an online class group), and students who accepted the above conditions completed the measurement tool over the weekend under the guidance of the counselors.

### Instruments

#### Physical activity rating scale (PARS-3)

The PARS-3 was introduced and revised by Liang [24] of Wuhan Sports University in China and has been widely used by researchers to measure students’ physical exercise levels [25]. It contains 3 items: (1) “Usually, your body changes after completing a physical exercise is?“ This question was used to test the exercise intensity of middle school students, and a 5-point Likert scale ranging from 1 (no sensation) to 5 (heavy sweating) was used; (2) “Usually, how many minutes do you last at a time when you perform the above physical exercise?“ This question was used to test the exercise time of middle school students, with a 5-point Likert scale ranging from 1 (less than 10 min) to 5 (more than 60 min) being used; (3) “Usually, how many times per month do you perform the above physical exercise? " This question was used to test the exercise frequency of middle school students, with a 5-point Likert scale ranging from 1 (less than 1 time a month) to 5 (approximately once a day) being used. The total physical exercise level score was calculated using the following formula: exercise intensity score × (exercise time score − 1) × exercise frequency score. In this study, Cronbach’s α coefficient for the overall PARS-3 was 0.72.

#### Student peer relationship scale (SPRS)

The SPRS developed by Asher et al. [26] and revised by Zhang [27] was used to test the level of middle school students’ peer relationships. It contains a total of 16 items, including three dimensions: welcome, e.g., “It’s easy for me to make new friends at school”; isolation, e.g., “I don’t have anyone to talk to”; and exclusion, e.g., “I don’t have anyone to play with.” The four-point Likert-type scale from 1 (strongly disagree) to 4 (strongly agree) was used. In this study, Cronbach’s α coefficient for the overall SPRS was 0.92 (welcome = 0.80, isolation = 0.84, and exclusion = 0.82).

#### Social anxiety Subscale of the self—consciousness scale (SASS-CS)

The SASS-CS developed by Fenigstein et al. [28] and revised by Xu et al. [29] was used to test the level of social anxiety of middle school students. It contains a total of 6 items, e.g., “I feel nervous when I speak in front of a crowd”. The four-point Likert-type scale from 1 (completely incompatible) to 4 (very compatible) was used. In this study, Cronbach’s α coefficient for the overall SASS-CS was 0.85.

#### Short (9-Item) dispositional Flow scales (SDFS-2)

The SDFS-2 developed by Jackson et al. [30] and revised by Liu [31] was used to test the flow experience in physical exercise of middle school students. It contains a total of 9 items corresponding to the 9 dimensions of the long (36-item) flow scales: Challenge Skill Balance: “I feel I am competent enough to meet the high demands of the situation”; Action-Awareness Merging: “I do things spontaneously and automatically without having to think”; Clear Goals: “I have a strong sense of what I want to do”; Unambiguous Feedback: “I have a good idea while I am performing about how well I am doing”; Concentration on Task at Hand: “I am completely focused on the task at hand”; Sense of Control: “I have a feeling of total control over what I am doing”; Loss Self-Consciousness: “I don’t worry about how others see me”; Transformation of Time: “The way time passes seems to be different from normal”; and Autotelic Experience: “This experience is extremely rewarding.” The five-point Likert-type scale from 1 (strongly disagree) to 5 (strongly agree) was used. In this study, Cronbach’s α coefficient for the overall SDFS-2 was 0.91.

### Statistical analyses

Various statistical analyses were conducted using SPSS 19.0, including descriptive and correlation analyses, independent-sample t-test, one-way ANOVA, and regression analysis. In addition, the Bootstrap detection method was used to test the moderated mediation model in PROCESS 3.5 [32]. Five thousand bootstrap resamples were set to calculate the 95% confidence intervals (*CI*) of the indirect effects in all statistical analyses. The significance level of all variables was set to α = 0.05.

## Results

As shown in Table 1, of the 1056 samples, 516 were male and 540 were female; 772 were rural and 284 were urban in origin; 316 were in the grade seven, 342 were in the grade eight, and 398 were in the grade nine. And in general, data conforming to a normal distribution have a skewness coefficient between − 3 and 3 and a kurtosis coefficient between − 10 and 10 [33]. The descriptive statistics (Table 2) indicated that all the measures had a relatively normal distribution with acceptable ranges of skewness (range = -0.71 to 1.51) and kurtosis scores (range = -0.65 to 3.24).

**Table 1:** Analysis of sociodemographic differences in study variables (N = 1056)

SV/RV	Physical exercise	Peer relationship	Social anxiety	Flow experience
Gender				
Male (N = 516)	33.49 ± 26.16	54.38 ± 7.47	11.80 ± 3.89	38.03 ± 6.41
Female (N = 540)	21.51 ± 29.30	53.56 ± 7.31	12.34 ± 4.64	34.97 ± 6.82
*T*	7.00***	1.81	-2.03*	7.51***
Origin				
Rural (N = 772)	25.66 ± 23.93	54.59 ± 7.53	11.89 ± 3.85	36.16 ± 6.37
Urban (N = 284)	27.59 ± 29.69	53.86 ± 7.34	12.03 ± 4.42	36.46 ± 6.89
*T*	-0.95	1.38	-0.44	-0.59
Education level				
GS (N = 316)^1^	33.93 ± 27.67	54.41 ± 6.73	12.38 ± 4.01	36.39 ± 5.96
GE (N = 342)^2^	34.30 ± 32.27	54.69 ± 8.12	11.16 ± 4.49	37.51 ± 7.07
GN (N = 398)^3^	16.76 ± 21.86	53.04 ± 7.24	12.54 ± 4.27	35.74 ± 7.11
*F*	51.06***	5.37***	10.60***	6.24***
LSD	3 < 1, 3 < 2	3 < 1, 3 < 2	2 < 3, 2 < 1	2 > 1, 2 > 3

SV, sociodemographic variables; RV, research variables; GS, grade seven; GE, grade eight; GN, grade nine

*** *p* < 0.001, * *p* < 0.05

**Table 2:** Descriptive statistics and correlation analysis of study variables

		1	2	3	4
1	Physical exercise	1			
2	Peer relationship	0.11***	1		
3	Social anxiety	-0.15***	-0.47***	1	
4	Flow experience	0.27***	0.39***	-0.25***	1
	Skewness	1.51	-0.28	0.57	-0.71
	Kurtosis	3.24	-0.65	-0.24	0.42
	Mean	27.41	53.97	12.07	36.48
	Standard deviation	28.42	7.40	4.30	6.79

***p < 0.001

Independent samples t-tests were used to test for gender differences and differences in origin for the study variables, the data passed the “Levene’s equality of variances test” (*p* > 0.05). In terms of gender, physical exercise scores and flow experience scores were significantly higher in boys than in girls (*p* < 0.05), and social anxiety scores were significantly lower in boys than in girls (*p* < 0.05). In terms of origin, there was no difference in origin for any of the four study variables (*p* > 0.05). One-way ANOVA was used to test for differences in the study variables in terms of education level, the data passed the “single-factor homogeneity test” and can use the one-way ANOVA (*p* > 0.05), and the results showed that the scores of physical exercise, peer relationships, social anxiety, and flow experience were significantly different in different educational level (*p* < 0.05). As a result, two variables, gender and educational level, were used as covariates in the following study.

Table 2 presents the results of the descriptive statistics and correlation analysis of the study variables. The correlation analysis showed that all the correlations between the variables were statistically significant. Physical exercise was positively correlated with peer relationships (*r* = 0.11, *p* < 0.001) and flow experience (*r* = 0.27, *p* < 0.001), and peer relationships were positively correlated with flow experience (*r* = 0.39, *p* < 0.001). Moreover, social anxiety was negatively correlated with physical exercise (*r* = -0.15, *p* < 0.001), peer relationships (*r* = -047, *p* < 0.001), and flow experience (*r* = -0.25, *p* < 0.001). The significant correlations between the study variables provided a good foundation for the testing of the subsequent study hypotheses.

Model 4 of the PROCESS macro was used to examine the mediating effect of peer relationships between physical exercise and social anxiety. All variables were first standardized and then a mediation analysis was conducted.

As shown in Table 3, first, with gender and education level as covariates, physical exercise as an independent variable, and social anxiety as a dependent variable, the regression coefficient of physical exercise on social anxiety was statistically significant (*b* = -0.15, *p* < 0.001, Model 1). Therefore, Hypothesis I was verified. Second, with gender and education level as covariates, physical exercise as an independent variable, and peer relationships as a dependent variable, the regression coefficient of physical exercise on peer relationships was statistically significant (*b* = 0.09, *p* < 0.01, Model 2). Third, with gender and education level as covariates, physical exercise and peer relationships as independent variables, and social anxiety as the dependent variable, both the regression coefficients of physical exercise on social anxiety (*b* = -0.11, *p* < 0.001, Model 3) and peer relationships on social anxiety (*b* = -0.47, *p* < 0.001, Model 3) were statistically significant.

**Table 3:** The relationship between physical exercise and social anxiety: moderated mediating effect

	Outcome							
	Social anxiety		Peer relationships		Social anxiety		Peer relationships	
	Model 1		Model 2		Model 3		Model 4	
	*b*	*t*	*b*	*t*	*b*	*t*	*b*	*t*
Predictors								
Gender	0.06	0.99	-0.07	-1.19	0.03	0.49	0.09	1.57
*Education level*	-0.02	-0.48	-0.07	-1.84	-0.05	-1.51	-0.09	-2.45*
*Physical exercise*	-0.15	-4.63***	0.09	2.63**	-0.11	-3.84***	-0.02	-0.51
*Peer relationships*					-0.47	-17.13***		
*Flow experience*							0.42	13.87***
Physical exercise * flow experience							0.11	3.39**
*R*^*2*^	0.16		0.13		0.49		0.41	
*ΔR*^*2*^	0.02		0.02		0.24		0.17	
*F*	8.75***		5.72***		81.73***		42.58***	

*** *p* < 0.001, ** *p* < 0.01

To further support the mediation effect, we performed the parametric bootstrapping procedure. The result showed that the 95% bias-corrected bootstrap confidence interval was between − 0.067 and − 0.013. These results indicated that peer relationships could mediate the relationship between physical exercise and social anxiety (indirect effect = -0.04). Therefore, Hypothesis II was verified.

**Fig. 2:**
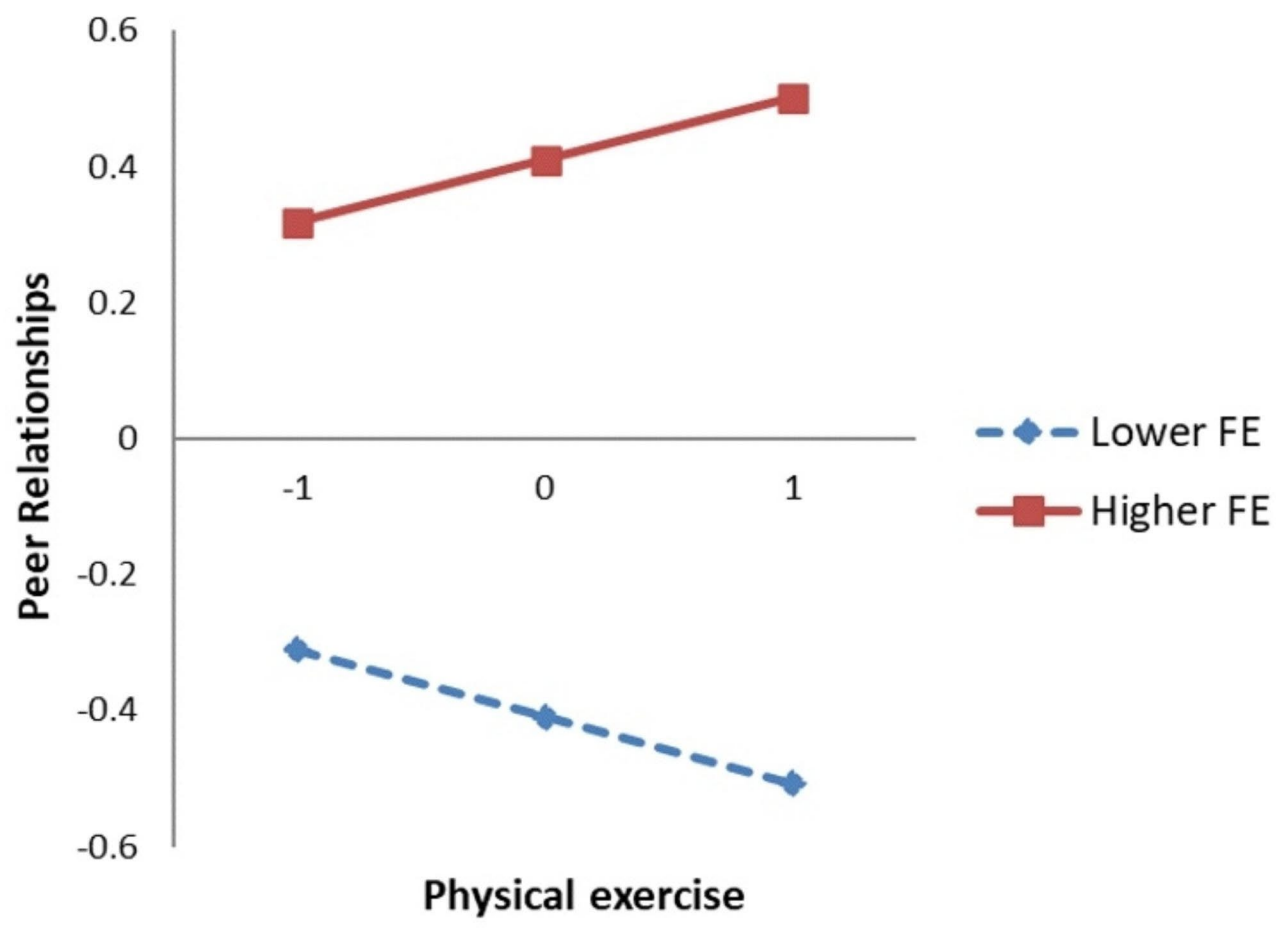
Moderating effect of flow experience on the relationship between Physical exercise and social anxiety FE, Flow experience

Model 7 of the PROCESS macro was used to test the moderated mediation model. The results of the linear regression analysis showed that the interaction between physical exercise and flow experience was significantly related to peer relationships (*b* = 0.11, *p* < 0.01, Model 4). Simple slope analysis was used to graphically illustrate the moderating effect of flow experience on the link between physical exercise and peer relationships. As shown in Fig. 2, physical exercise was negatively related to peer relationships when the score of flow experience was low (*b* = -0.12, *t* = -2.69, *p* < 0.01), and physical exercise was positively related to peer relationships when the score of flow experience was high (*b* = 0.09, *t* = 2.17, *p* < 0.05). Thus, H III was supported.

Specifically, for the mediating effect of peer relationships between physical exercise and social anxiety, under low-flow experience, peer relationships played a competitive partial mediating role between physical exercise and social anxiety (indirect effect = 0.06, 95% *CI* = [0.016, 0.105]), and under high-flow experience, peer relationships played a complementary partial mediating role between physical exercise and social anxiety (indirect effect = -0.04, 95% *CI* = [-0.087, -0.003]).

## Discussion

The current study proposed and tested a moderated mediation model to examine the relationships among physical exercise, peer relationships, flow experience, and social anxiety in a sample of Chinese middle school students. The findings of the present model are consistent with our hypotheses. Physical exercise could negatively predict social anxiety, and peer relationships mediated the relationship between physical exercise and social anxiety. We also found that flow experience moderated the relationship between physical exercise and peer relationships.

### The effect of physical exercise on social anxiety

Physical exercise has received considerable attention as a means of improving social anxiety. Previous studies have shown that individuals can develop positive psychological qualities during participation in physical exercise, which can reduce the level of social anxiety [34]. In the specific intervention operation, Jazaieri et al. [35] conducted an 8-week Mindfulness-Based Stress Reduction (MBSR) and aerobic exercise intervention for individuals with social anxiety. They found that both MBSR and aerobic exercise were equally effective in reducing social anxiety. However, aerobic exercise was a more effective intervention for individuals with high levels of social anxiety. In addition, different forms of physical exercise, such as basketball, volleyball, and physical dance, have been shown to reduce social anxiety in students [5]. The evidence presented above supports the negative relationship between physical exercise and social anxiety, as well as the causal relationship between the two. Through a cross-sectional study, we further supported the negative relationship between physical exercise and social anxiety in middle school students, providing a practical reference for improving social anxiety in middle school students through physical exercise.

### The mediating role of peer relationships between physical exercise and social anxiety

Several studies have shown that peer relationships are a crucial mediating variable between physical exercise and physical exercise outcome variables [18, 36]. And the present study also proposed and tested the hypothesis that physical exercise would affect social anxiety in middle school students through the mediating role of peer relationships. This pathway can be explained by the following two aspects. On the one hand, participation in physical exercise expands the social network of student groups, enhancing the heterogeneity and breadth of the social network [36]. It is beneficial for individuals to generate social capital, build social confidence, and improve peer relationships while participating in physical exercise [37]. On the other hand, peer relationships play an important role in the development of functions related to the early adolescent self-system, providing essential external information for constructing and maintaining core self-evaluation [8]. The cognitive model of social anxiety mentions that negative self-evaluation is the main cause of social anxiety [7]. Therefore, peer relationships can influence core self-evaluation and thus prevent the development of social anxiety [38]. As a result, engaging in physical exercise can offer more opportunities for middle school students to communicate, develop peer relationships, form positive self-evaluations, become less concerned about making a bad impression during future interactions, and ultimately avoid the emergence of social anxiety.

### The moderating role of flow experience between physical exercise and peer relationships

In the previous discussion, it was mentioned that physical exercise could offer middle school students more opportunities to communicate with their peers and foster positive peer relationships. However, participation in physical exercise alone may not be sufficient to improve individuals’ levels of peer relationships. Rather, it is likely related to their experiences during physical exercise [10]. **Hence**, we hypothesized that the flow experience, representing ideal experiential states, might moderate the impact of physical exercise on peer relationships. The results also confirmed our hypothesis that physical exercise was positively related to middle school students’ peer relationships at high levels of flow experience, whereas physical exercise was negatively related to middle school students’ peer relationships at low levels of flow experience. The reason for this phenomenon may be due to the fact that middle school students with high levels of flow experience are able to highly focus their attention highly on physical exercise and have a high sense of control over the current exercise task, which helps them to have excellent performance in interpersonal interactions, thus strengthening the trust of their exercise partners [12] and contributing to the establishment of peer relationships. However, if middle school student’s level of flow experience during physical exercise is too low, they are more likely to view physical exercise as a burden, and even though physical exercise gives them the opportunity to interact with their peers, they are more likely to participate in physical exercise with negative emotions, which is detrimental to the establishment of peer relationships. As noted by Xu et al. [39], positive exercise experiences can trigger positive emotions and foster peer relationships, whereas negative exercise experiences can exacerbate psychological distress and hinder the development of peer relationships.

### Implication

Social anxiety has become a common psychological disorder among middle school students, which seriously affects their healthy physical and mental development [40]. The current study proposed and tested a moderated mediation model to examine the relationships among physical exercise, flow experience, peer relationships, and social anxiety in a sample of Chinese middle school students, providing implications for the suppression of social anxiety in middle school students.

Our study confirmed that physical exercise was negatively related to social anxiety in middle school students and that peer relationships played a mediating role between physical exercise and social anxiety. Therefore, we recommend that the Chinese government continues to promote and invest in national fitness strategies to raise awareness of physical exercise among middle school students. This will encourage students to actively participate in physical exercise [41], thereby reducing social anxiety. To enhance the effectiveness of physical exercise interventions for social anxiety, educators should focus on fostering positive peer relationships among students during their participation in physical exercise. One way to achieve this goal is to incorporate group sports such as basketball, soccer, and volleyball, which facilitate stronger peer connections [5].

In addition, the moderating role of flow experience between physical exercise and peer relationships in middle school students suggests that educators should not only encourage their students to actively participate in physical exercise, but also pay attention to the acquisition of flow experience during their physical activities. This can be achieved by referring to Liu et al. [19], who set reasonable exercise goals for students during their exercise routines.

### Limitations and future research

As with any study, our study is not without its limitations. First, the research design was cross-sectional in nature, which limited our ability to allow for predictions of causality. In the future, causal relationships among study variables could be further tested using longitudinal designs and experimental studies. Second, all surveys involved self-report, so the common methodological bias may have affected the results, future studies should collect data from multiple informants (e.g., parents, teachers, or peers) to make the findings more persuasive. Third, this study only examined the important role of flow experience and peer relationships between physical exercise and social anxiety. In the future, more variables that may have a mediating role, e.g., physical self-esteem, core self-evaluation, etc., should be considered to improve research in this area. Finally, participants consisted only of Chinese middle school students, it is uncertain whether the cultural context of Chinese students is generalizable to the wider population. In the future, samples from different countries should be collected to test the applicability of the model in different cultural contexts.

## Conclusion

The present study is unique in proposing a moderated mediation model to explain the association between physical exercise and social anxiety and found that peer relationships mediate the relationship between physical exercise and social anxiety. We also found that the flow experience would moderate the relationship between physical exercise and peer relationships, only under high-flow experience, peer relationships would play a complementary partial mediation role between physical exercise and social anxiety. This study further expands the path of physical exercise affecting social anxiety, provides evidence for clarifying the relationship between physical exercise and social anxiety, and provides a practical reference for inhibiting middle school students’ social anxiety.

## Data Availability

The datasets used and/or analyzed during the current study are available from the corresponding author on reasonable request.
